# Performance of a quality control center supporting national antimicrobial resistance surveillance

**DOI:** 10.3389/bjbs.2026.16457

**Published:** 2026-06-03

**Authors:** Dong Woo Shin, Hyunji Kim, Jeong Su Park, Kyoung Un Park, Min Hyuk Choi, Dokyun Kim, Seok Hoon Jeong, Hee Jung Kim, Young Ah Kim, Kwangjin Ahn, Young Uh, Yong Jun Kwon, Jong Hee Shin, Soo Hyun Kim, Jeong Hwan Shin, Hee Young Kang, Dong Chan Moon, Sung Young Lee, Songmee Bae

**Affiliations:** 1 Department of Laboratory Medicine, Seoul National University Bundang Hospital, Seongnam, Republic of Korea; 2 Department of Laboratory Medicine, Seoul National University College of Medicine, Seoul, Republic of Korea; 3 Department of Laboratory Medicine and Research Institute of Bacterial Resistance, Yonsei University College of Medicine, Seoul, Republic of Korea; 4 Department of Laboratory Medicine, National Health Insurance Service, Ilsan Hospital, Goyang, Republic of Korea; 5 Department of Laboratory Medicine, Yonsei University Wonju College of Medicine, Wonju, Republic of Korea; 6 Department of Laboratory Medicine, Chonnam National University Medical School, Gwangju, Republic of Korea; 7 Department of Laboratory Medicine and Paik Institute for Clinical Research, Inje University College of Medicine, Busan, Republic of Korea; 8 Division of Antimicrobial Resistance Research, National Institute of Health, Korea Disease Control and Prevention Agency, Cheongju-si, Republic of Korea

**Keywords:** antimicrobial resistance, antimicrobial susceptibility testing, external quality assessment, interlaboratory proficiency testing, quality assurance

## Abstract

**Introduction:**

Ensuring the reliability, standardization, and international comparability of antimicrobial resistance (AMR) surveillance data critically depends on the implementation of robust quality assurance frameworks. South Korea established the Korea Global Antimicrobial Resistance Surveillance System (Kor-GLASS), supported by a centralized quality control center (QCC). As Kor-GLASS transitioned from Phase II to Phase III, new bacterial species and antimicrobial agents were incorporated, underscoring the need to evaluate whether quality assurance performance could be sustained during system expansion.

**Materials and Methods:**

We analyzed interlaboratory proficiency testing (IPT) and external quality assessment (EQA) outcomes generated by the QCC between 2020 and 2024, covering Phases II and III of Kor-GLASS. Clinical isolates were collected at participating hospitals and transferred to organism-specialized analysis centers for standardized antimicrobial susceptibility testing (AST), while the QCC independently oversees data quality through IPT and EQA. IPT was conducted by comparing AST results between analysis centers and the QCC using subsets of routine clinical isolates, with acceptance criteria defined as categorical agreement (CA) ≥90% and major error rates <3%. EQA involved quarterly distribution of pre-characterized strains to participating centers. Additional evaluations addressed the performance of newly introduced ceftazidime-avibactam susceptibility testing and interlaboratory validation for *Haemophilus* spp.

**Results:**

Across the study period, overall CA consistently exceeded 97% in IPT, and no EQA failures observed among participating centers. While major errors during Phase II were primarily attributable to AST reading and near-breakpoint discrepancies, their frequency markedly decreased in Phase III following targeted corrective actions and educational interventions. Susceptibility testing for ceftazidime-avibactam showed high concordance between centers, with rare discrepancies limited to near-breakpoint measurements. Interlaboratory validation confirmed acceptable performance for AST of *Haemophilus* spp., supporting its formal inclusion in Phase III.

**Discussion:**

These findings demonstrate that a centralized, QCC-led quality assurance framework can maintain stable and reliable AMR surveillance performance during periods of system expansion. Beyond routine oversight, coordinated quality assurance activities function as an evidence-based evaluation of how standardized laboratory data are generated and validated, reinforcing their essential role in sustaining the credibility and future development of AMR surveillance systems.

## Introduction

Ensuring the reliability, standardization, and international comparability of laboratory-derived data, such as antimicrobial resistance (AMR) surveillance data, fundamentally depends on the implementation of robust quality assurance processes [1, 2]. In response to the growing global burden of AMR, the World Health Organization (WHO) launched the Global Antimicrobial Resistance and Use Surveillance System (GLASS) in 2015 as an international framework for standardized AMR surveillance [1]. Within this global context, South Korea established the Korea Global Antimicrobial Resistance Surveillance System (Kor-GLASS) in 2016 to generate high-quality, standardized AMR data compatible with GLASS [3], with support from an independent quality assurance framework.

**FIGURE 1:**
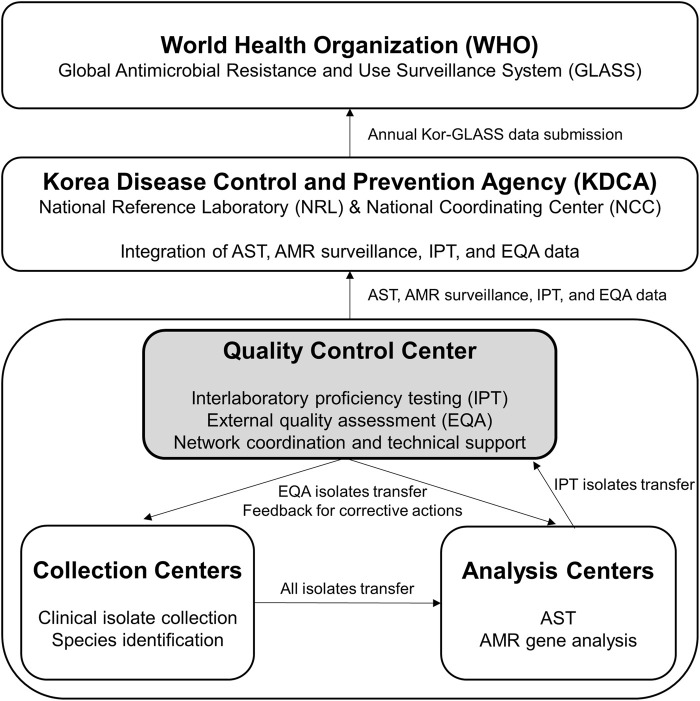
Operational framework of the Kor-GLASS network centered on the Quality Control Center. Clinical isolates are collected and identified at collection centers and subsequently transferred to species-specialized analysis centers for antimicrobial susceptibility testing (AST) and antimicrobial resistance (AMR) gene analysis. The Quality Control Center (QCC) conducts interlaboratory proficiency testing (IPT) using a subset of collected isolates and performs external quality assessment (EQA) by distributing pre-characterized isolates to participating centers, with feedback provided accordingly. Results of IPT and EQA are reported to the Korea Disease Control and Prevention Agency (KDCA). Aggregated Kor-GLASS outputs, including nationwide AST results generated across the network, are submitted annually to the WHO Global Antimicrobial Resistance and Use Surveillance System (GLASS).

The Kor-GLASS network consists of three key components: collection centers, analysis centers, and a quality control center (QCC) (Figure 1). In this framework, clinical isolates identified at collection centers are forwarded to designated analysis centers according to predefined organism group-based specialization. Each analysis center performs standardized antimicrobial susceptibility testing (AST) for its assigned pathogen groups, while the QCC oversees network-wide data quality and harmonization. Over successive project phases, the network has been progressively expanded and currently comprises 13 university hospitals nationwide, ensuring broad geographic coverage and representativeness of antimicrobial resistance surveillance across Korea.

The QCC performs quality assurance of the collected AMR data through interlaboratory proficiency testing (IPT) and external quality assessment (EQA) [4]. IPT evaluates the concordance of AST results between analysis centers and the QCC using a subset of collected isolates, whereas EQA assesses laboratory performance through the distribution of pre-characterized strains for species identification, AST, and AMR gene analysis. In addition to its role in the Kor-GLASS network, the QCC supports the One Health project in Korea by providing quality assurance for participating institutions, managing reference strain deposits, and conducting educational programs. Through these quality assurance activities, the QCC plays a key role in supporting data validity and comparability across the AMR surveillance network.

Kor-GLASS has progressed through Phase I (2017–2019), Phase II (2020–2022), and is currently in Phase III (2023–2025). Notable Phase III updates include the addition of new antibiotics for susceptibility testing (ceftazidime-avibactam for Enterobacterales) and the inclusion of additional bacterial species (*Haemophilus* spp.). This study aims to analyze and compare the quality assurance outcomes generated during Phases II and III, with a particular focus on updates introduced in Phase III, summarize the QCC’s activities, and discuss the importance and future direction of quality assurance in national AMR surveillance.

## Materials and methods

### Role of collection centers and analysis centers

As of Phase III, the Kor-GLASS network includes 13 university hospitals; among these, five function as collection centers, seven as analysis centers (five of which also serve as collection centers), and one as the QCC. Within the Kor-GLASS framework, clinical isolates obtained at collection centers are transferred to designated analysis centers for standardized identification, AST, and AMR gene analysis. Isolates are shipped in 10% skim milk preservation medium prepared by dissolving commercial skim milk powder (MBCell, KisanBio, Seoul, South Korea) in distilled water, to ensure consistency during transport and processing. As of Phase III, seven analysis centers are actively participating in the network, collectively covering 15 target pathogens. Each analysis center specializes in one or more predefined organism groups, including *Staphylococcus aureus*, *Enterococcus faecalis*, *Enterococcus faecium*, *Neisseria gonorrhoeae*, *Salmonella* spp., *Shigella* spp., *Streptococcus pneumoniae*, *Haemophilus* spp., *Neisseria meningitidis*, *Acinetobacter* spp., *Pseudomonas aeruginosa*, *Candida* spp., *Clostridioides difficile*, *Escherichia coli*, and *Klebsiella pneumoniae*. The bacterial species, AST panels, and AMR genes assigned to each analysis center are summarized in Supplementary Table S1.

Kor-GLASS was developed in alignment with the WHO GLASS framework, including surveillance principles, target pathogens, and antimicrobial-pathogen combinations [5]. All participating laboratories performed laboratory procedures in accordance with the standardized Kor-GLASS operating procedures. These procedures incorporated Clinical and Laboratory Standards Institute (CLSI)-based laboratory methods and interpretive criteria, including guidance on disk diffusion [6], broth microdilution [7], AST breakpoints [8, 9], and microbial identification by targeted DNA sequencing [10]. Bacterial species identification was primarily conducted using matrix-assisted laser desorption/ionization time-of-flight mass spectrometry (MALDI-TOF MS). When species-level identification was not achieved or confirmatory identification was required, gene sequencing methods including 16S rRNA and *rpoB* sequencing for bacterial isolates and internal transcribed spacer (ITS) sequencing for *Candida* spp. were performed. AST was carried out using either the broth microdilution method or the disk diffusion method, depending on the bacterial species and antimicrobial agents tested. For *Clostridioides difficile*, AST was performed using the agar dilution method, which differs substantially from routine AST methods used for other target species, and was not included in IPT by the QCC. AMR genes were analyzed using polymerase chain reaction (PCR)-based methods or sequencing techniques.

For clinical breakpoints, CLSI M100 30th edition [8] was applied throughout Phase II (2020–2022), whereas CLSI M100 33rd edition [9] was applied throughout Phase III (2023–2025), to maintain consistency within each surveillance phase.

### Interlaboratory proficiency testing (IPT)

Within the Kor-GLASS framework, IPT refers to an interlaboratory comparison and retesting process in which the QCC independently retests a subset of routine clinical isolates submitted by analysis centers. IPT was generally conducted on a monthly basis. For organism groups with a limited number of isolates, including *Salmonella* spp., *Streptococcus pneumoniae*, *Candida* spp., and *Haemophilus* spp., IPT was performed at a reduced frequency.

IPT was performed by comparing AST results between the analysis centers and the QCC. Approximately 5% of isolates from each analysis center were randomly selected during each IPT round; when the total number of isolates was limited, a minimum of five isolates was selected. The QCC independently performed AST on the selected and transferred isolates, and the results were compared with those generated by the corresponding analysis center.

For practical purposes within the Kor-GLASS scheme, both susceptible–resistant (S–R) and resistant–susceptible (R–S) discordances were counted together as major errors, without separately applying the conventional very major error category. Minor errors were defined as discrepancies involving intermediate (I) interpretations (S–I or I–R). Categorical agreement (CA) was defined as concordant classification into susceptible, intermediate, or resistant categories between the two centers. An IPT result was considered acceptable when the major error rate was <3% and the categorical agreement was ≥90%. Antifungal susceptibility testing for *Candida* spp. was assessed using CA, essential agreement (within ±2 dilutions of the minimum inhibitory concentration), and total agreement, in accordance with established antifungal susceptibility testing metrics [11].

In cases involving major errors or failure to meet the acceptance criteria, the analysis center was required to submit a corrective action report, including an investigation of the underlying causes and implementation of appropriate corrective measures.

In this study, IPT results obtained during 2020–2024 (Phase II and the ongoing Phase III) were analyzed.

### External quality assessment (EQA)

Within the Kor-GLASS framework, EQA refers to an assessment of the performance of participating centers based on test results for pre-characterized strains distributed by the QCC. EQA was conducted quarterly by distributing pre-characterized strains from the QCC to collection centers and analysis centers. Each EQA round typically consisted of five strains and was designed to evaluate performance in species identification, AST, and AMR gene analysis, according to the role of each participating center. Collection centers performed species identification only. Analysis centers performed species identification for all distributed strains, while AST and AMR gene analysis were conducted exclusively for strains belonging to the organism groups assigned to each analysis center.

The results of species identification, AST, and AMR gene analysis were independently evaluated and scored as percentage agreement. An overall score of ≥90% was required to achieve a pass status. In cases where the acceptance criteria were not met, the participating center was required to investigate the causes of discordant results and submit a corrective action report detailing the identified issues and implemented remedial measures.

In this study, EQA results obtained during 2020–2024 (Phase II and the ongoing Phase III) were analyzed.

### IPT performance of newly introduced ceftazidime-avibactam

Following the addition of ceftazidime-avibactam to the AST panel in the Kor-GLASS Phase III, its analytical performance was further evaluated as part of routine IPT. AST for ceftazidime-avibactam was performed using the disk diffusion method for *Escherichia coli* and *Klebsiella pneumoniae*. Results generated at the designated analysis center were compared with those independently obtained by the QCC to assess CA and differences in zone diameter measurements.

### Interlaboratory validation for a newly introduced bacterial species

To support the introduction of *Haemophilus* spp. as an additional target bacterial species within the Kor-GLASS Phase III analytical framework, an interlaboratory validation was conducted prior to formal implementation. An existing analysis center, previously designated for *Salmonella* spp., *Shigella* spp., *Streptococcus pneumoniae*, and *Neisseria meningitidis*, participated in this validation as a candidate center for the expansion of its analytical scope to include *Haemophilus* spp.

AST results for validation isolates of *Haemophilus* spp. generated by the candidate analysis center were compared with results independently generated by two separate teams within the National Reference Laboratory (NRL teams I and II), and the QCC. Following three pilot rounds, a formal validation round was conducted, in which predefined acceptance criteria (major error rate <3% and categorical agreement ≥90%, based on pairwise comparisons against the QCC) were met, thereby confirming the suitability of the expanded analytical scope.

### Additional activities of the QCC

In addition to IPT and EQA, the QCC undertook supplementary quality assurance activities to support sustained standardization, coordination, and capacity building within the Kor-GLASS network and the national One Health framework. These activities included regular communication with Kor-GLASS participating institutions, as well as quality control support for laboratories participating in the Korean One Health project that perform AST of non-human-derived isolates. The QCC also provided technical consultation and educational support. Educational and feedback activities were delivered through online practical lectures for laboratory technicians, quarterly Kor-GLASS operational meetings, and feedback accompanying IPT and EQA reports, with more focused support provided when new centers, target organisms, or antimicrobial agents were introduced. In addition, the QCC deposited reference strains to support standardized laboratory practice. All QCC activities related to culture, preservation, IPT, and EQA for antimicrobial-resistant organisms were conducted under an ISO 9001:2015-certified quality management system.

### Statistical analysis

Interlaboratory agreement of ceftazidime-avibactam disk diffusion zone diameter measurements between the analysis center and the QCC was evaluated using Bland-Altman analysis [12] with Microsoft Excel (Microsoft Corp., Redmond, WA, USA). The mean zone diameter and interlaboratory difference (analysis center - QCC) were calculated for each paired measurement. The mean difference, standard deviation of the differences, and upper and lower 95% limits of agreement were then calculated. The results were visualized as a Bland-Altman plot using the scatter plot function. Exact McNemar test was performed using SPSS software version 27 (IBM Corp., Armonk, NY, USA) to assess directional discordance between susceptible/resistant classifications.

## Results

### IPT results

**FIGURE 2:**
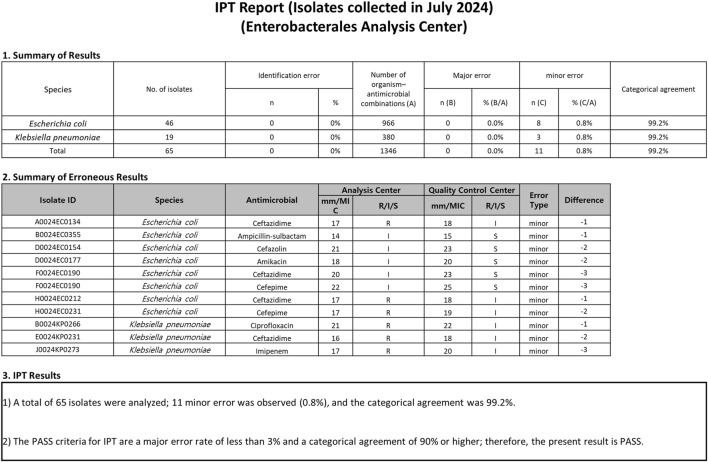
Representative interlaboratory proficiency testing (IPT) report. A representative summary page of an interlaboratory proficiency testing (IPT) report. The report includes information on the IPT round, overall error rates, and categorical agreement for antimicrobial susceptibility testing. Erroneous results identified during the IPT round are summarized.

A representative IPT report is presented in Figure 2, and the aggregated IPT results are summarized in Table 1. During Phase II (2020–2022), a total of 180 IPT reports were generated based on 2,381 isolates, whereas 122 IPT reports were issued from 1,291 isolates during Phase III (2023–2024; ongoing). For *Candida* spp., during Phase II, 63 isolates were evaluated, yielding CA, essential agreement, and total agreement rates of 99.8%, 96.8%, and 98.3%, respectively; in Phase III, 49 isolates were assessed, with corresponding agreement rates of 99.7%, 100%, and 99.9%. *Shigella* spp., *Neisseria meningitidis*, and *Neisseria gonorrhoeae* were not included in the IPT due to extremely limited availability of eligible clinical isolates. Across all species tested, the overall categorical agreement consistently exceeded 97% throughout the study period, and this performance was maintained across the transition from Phase II to Phase III.

**TABLE 1:** Interlaboratory proficiency testing (IPT) results between 2020 and 2024.

​	2020–2022 (Phase II)				2023–2024 (Phase III; ongoing)			
Species	n	ME	mE	CA	n	ME	mE	CA
EC	1464	0.2%	2.9%	97.1%	455	0.1%	1.7%	98.3%
KP	367	0.4%	2.2%	97.8%	415	0.1%	1.2%	98.8%
SA	161	0.2%	0.9%	99.1%	120	0.0%	0.1%	99.9%
EFM	94	0.3%	2.5%	97.5%	72	0.9%	2.8%	97.2%
EFA	62	0.0%	2.5%	97.5%	48	1.0%	2.2%	97.8%
AC	103	0.4%	3.8%	96.2%	65	0.2%	2.3%	97.7%
PA	69	0.4%	4.7%	95.3%	55	0.0%	5.2%	94.8%
SL	40	0.0%	1.1%	98.9%	25	0.0%	0.9%	99.1%
SP	21	0.3%	10.2%	89.5%	25	0.4%	5.2%	94.5%
HI	-	-	-	-	11	0.0%	1.3%	98.7%
Total	2381	0.2%	2.8%	97.2%	1291	0.2%	1.7%	98.3%

EC, *Escherichia coli*; KP, *Klebsiella pneumoniae*; SA, *Staphylococcus aureus*; EFM, *Enterococcus faecium*; EFA, *Enterococcus faecalis*; AC, *Acinetobacter* spp.; PA, *Pseudomonas aeruginosa*; SL, *Salmonella* spp.; SP, *Streptococcus pneumoniae*; HI, *Haemophilu*s spp.; ME, major error; mE, minor error; CA, categorical agreement.

### EQA results

**FIGURE 3:**
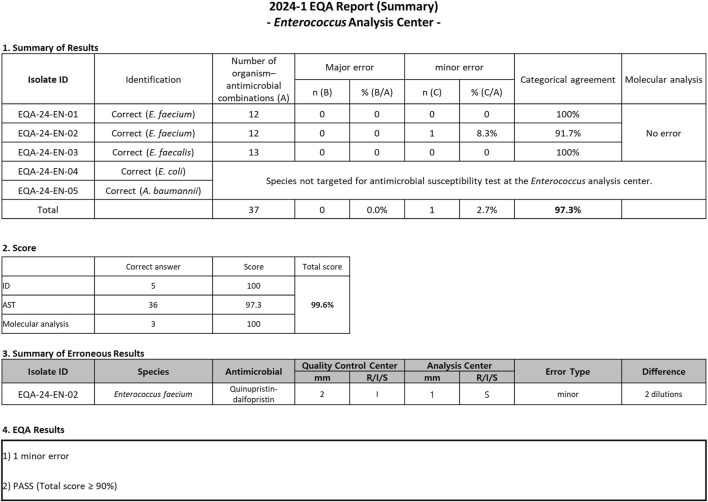
Representative external quality assessment (EQA) report. A representative EQA report selected from the actual reports is shown. The report summarizes the results of species identification, antimicrobial susceptibility testing (AST), and antimicrobial resistance (AMR) gene analysis for five pre-characterized strains. Erroneous results identified during the EQA round are indicated, and the overall pass/fail status based on the composite performance score is presented.

Figure 3 shows a representative EQA report selected from the actual reports issued during the study period, whereas Table 2 summarizes the overall EQA results of all participating centers. During the 5-year period, 226 EQA reports were generated from 12 participating centers, consisting of five collection centers and seven analysis centers; one collection center joined the EQA program from the third quarter of 2023. Overall, all participating centers demonstrating sustained compliance with predefined quality criteria, with no EQA failures observed.

**TABLE 2:** External quality assessment (EQA) results of participating centers (2020–2024).

Center	Score (%)^a^				
​	2020	2021	2022	2023	2024
ECKP	99.5	98.8	99.0	99.8	99.3
SA	100	100	100	100	100
EN	99.4	99.7	99.5	99.5	99.4
ACPA	98.3	99.3	100	99.8	98.3
SLSP	100	99.3	98.0	98.8	98.9
CN	99.8	100	99.5	100	100
CD	100	100	100	100	100
CC1	100	100	100	100	100
CC2	100	100	100	100	100
CC3	100	100	100	100	100
CC4	100	100	100	100	100
CC5^b^	-	-	-	100	100

^a^ Scores represent the annual mean of four quarterly external quality assessment (EQA) rounds conducted each year.

^b^ One collection center joined the EQA, program from the third quarter of 2023; therefore, the total number of EQA reports during the study period was 226 rather than 240.

EC, *Escherichia coli*; KP, *Klebsiella pneumoniae*; SA, *Staphylococcus aureus*; EFM, *Enterococcus faecium*; EFA, *Enterococcus faecalis*; AC, *Acinetobacter* spp.; PA, *Pseudomonas aeruginosa*; SL, *Salmonella* spp.; SP, *Streptococcus pneumoniae*; HI, *Haemophilus* spp.; CN, *Candida* spp.; CC, collection center.

### Corrective actions for major errors identified in IPT and EQA

The IPT pass rate was 100% during Phase II (2020–2022) and 96.2% during Phase III (2023–2024; ongoing). The EQA pass rate was 100% throughout the study period. For IPT and EQA rounds that did not meet the pass criteria, as well as for rounds that passed but contained major errors, appropriate corrective actions were implemented according to the identified causes (Table 3). During Phase II, a total of 26 major errors were identified, with reading-related errors and near-breakpoint discrepancies accounting for the majority of cases. These errors were primarily associated with difficulties in zone interpretation, disk placement, and narrow interpretive breakpoints. In contrast, the number of major errors decreased to four cases during Phase III, all of which were attributable to data entry errors or sample-related issues. Notably, no reading-related errors were observed during Phase III following targeted educational interventions, indicating a substantial improvement in interpretive consistency.

**TABLE 3:** Summary of major errors in IPT and corresponding corrective actions by study period.

Cause category	Corrective action	No. of cases	
​	​	2020–2022	2023–2024
Reading error - Double rim or fuzzy growth - Inadequate disk adhesion - Antibiotic-specific reading rule - Disk misalignment during reading - Misinterpretation of interpretive category	Education conducted	9	0
Near-breakpoint discrepancy - Interpretative category differed due to narrow breakpoint	Repeat testing performed	9	0
Data entry error - Data input - Spreadsheet logic	Data entry form reviewed Spreadsheet logic corrected	3	2
Sample-related error - Contaminated isolates - Mixed isolates	Sample re-shipped Repeat testing performed	2	2
Unexplained discrepancy	Repeat testing performed	3	0

### Performance of IPT for newly introduced ceftazidime-avibactam

**FIGURE 4:**
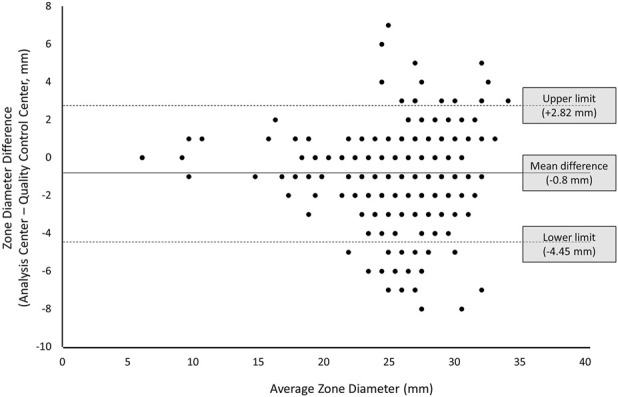
Bland-Altman plot for interlaboratory agreement of ceftazidime-avibactam zone diameter measurements. The plot shows the agreement of disk diffusion zone diameter measurements between the analysis center and the quality control center (QCC) for Enterobacterales isolates. The solid line represents the mean difference (analysis center - QCC), and the dashed lines indicate the 95% limits of agreement.

Following the introduction of ceftazidime-avibactam into the AST panel in Phase III (2023–), disk diffusion results were analyzed for a total of 870 Enterobacterales isolates. Complete categorical agreement was observed for 455 *Escherichia coli* isolates, whereas three discordant results (0.7%) were identified among 415 *Klebsiella pneumoniae* isolates (Table 4). Exact McNemar testing showed no statistically significant directional difference in susceptible and resistant classifications of *K. pneumoniae* between the analysis center and the QCC (p = 0.25). All discordant cases represented near-breakpoint discrepancies, in which zone diameters measured at the analysis center were 19–20 mm (interpreted as resistant), while corresponding measurements at the QCC ranged from 22 to 26 mm (interpreted as susceptible), based on the breakpoint definition (≤20 mm, resistant; ≥21 mm, susceptible) [9]. The mean zone diameters and distribution ranges for susceptible and resistant isolates were highly comparable between the two centers across both species. In addition, Bland–Altman analysis demonstrated a small interlaboratory bias in zone diameter measurements between the analysis center and the QCC (mean difference [analysis center - QCC], −0.81 mm) (Figure 4).

**TABLE 4:** Comparison of antimicrobial susceptibility testing results for ceftazidime-avibactam in Enterobacterales between two centers.

Species	​	Analysis center			Quality control center		
​	​	n	ZD (mean ± SD)	ZD (range)	n	ZD (mean ± SD)	ZD (range)
*Escherichia coli*	S	448	26.8 ± 2.1	21–34	448	27.5 ± 2.1	21–32
(n = 455)	R	7	10.9 ± 4.9	6–20	7	11.0 ± 5.0	6–20
*Klebsiella pneumoniae*	S	361	24.6 ± 2.5	21–35	364	25.6 ± 2.5	21–35
(n = 415)	R	54	17.6 ± 3.4	6–20	51	17.7 ± 3.7	6–20
Total	S	809	25.8 ± 2.5	21–35	812	26.5 ± 2.5	21–35
(n = 870)	R	61	16.7 ± 4.2	6–20	58	16.9 ± 4.4	6–20

ZD, zone diameter; SD, standard deviation; S, susceptible; R, resistant.

### Interlaboratory validation of AST for *Haemophilus* spp.

Interlaboratory validation results of AST for *Haemophilus* spp. are summarized in Table 5. Following completion of the three pilot rounds, a formal validation round was performed, in which all predefined acceptance criteria were met based on pairwise comparisons against the QCC. These results confirmed the suitability of expanding the analytical scope of the existing analysis center to include *Haemophilus* spp.

**TABLE 5:** Interlaboratory validation results for a newly introduced bacterial species (*Haemophilus* spp.).

Round	Center	ME	mE	CA
Pilot-1	NRL team I	0%	11.1%	88.9%
​	NRL team II	5.6%	13.3%	86.7%
​	Analysis center	0%	13.3%	86.7%
Pilot-2	NRL team I	3.3%	5.6%	94.4%
​	NRL team II	4.4%	2.2%	97.8%
​	Analysis center	1.1%	4.4%	95.6%
Pilot-3	NRL team I	0%	5.6%	94.4%
​	NRL team II	0%	5.6%	94.4%
​	Analysis center	0%	4.4%	95.6%
Formal round	NRL team I	0%	6.7%	93.3%
​	NRL team II	1.1%	4.4%	95.6%
​	Analysis center	0%	1.1%	98.9%

ME, major error; mE, minor error; CA, categorical agreement; NRL, National Reference Laboratory.

### Additional activities of the QCC within the one health framework

During the study period, the QCC provided quality control support and educational programs to six additional laboratories participating in the Korean One Health project. Network coordination was maintained through quarterly Kor-GLASS meetings, and annual educational programs were conducted to reinforce standardized practices across sectors.

Furthermore, seven rare and epidemiologically significant strains, including multi-carbapenemase-producing Enterobacterales, were fully characterized and deposited as reference isolates in the National Culture Collection for Pathogens. Throughout the study period, the QCC maintained its ISO 9001:2015-certified quality management system.

## Discussion

Quality assessment involves the collection and analysis of data to evaluate the extent to which predefined standards and criteria are met, whereas quality assurance encompasses continuous, system-level activities designed to promote and maintain quality through the establishment and application of such standards [13]. In the present study, IPT and EQA served as key components supporting broader quality assurance activities within the surveillance system. This study provides a comprehensive evaluation of the quality assurance performance of the QCC during the transition from Kor-GLASS Phase II to Phase III. Despite the introduction of an additional bacterial species and a newly incorporated antimicrobial agent, IPT continued to show consistently high categorical agreement. No EQA failures were observed during the study period, indicating that the composite scores integrating species identification, AST, and AMR gene analysis met the predefined passing criterion, rather than indicating flawless concordance across all tested items. Although a causal relationship was not formally established, the reduction in major errors attributable to reading-related and interpretive issues was observed following systematic corrective actions and targeted educational interventions coordinated by the QCC. Together, these findings demonstrate that a centralized, QCC-led integrated quality assurance framework can support reliable and comparable AMR data generation during periods of system transition, reinforcing the role of structured quality assurance mechanisms in national surveillance. In this context, the present study conceptualizes centrally coordinated quality assurance activities not merely as operational oversight, but as a research-driven evaluation of how standardized antimicrobial susceptibility data are generated, validated, and transformed into public health surveillance outputs at the interface of clinical laboratory practice and national public health governance.

In the Kor-GLASS framework, IPT represents a distinctive quality assurance activity coordinated by the QCC that supports the reliability of multiple studies derived from Kor-GLASS-generated AMR data. Unlike conventional EQA schemes that rely on pre-characterized reference strains selected specifically for proficiency testing, IPT in Kor-GLASS is performed using a subset of routine clinical isolates collected for national AMR surveillance. Through this mechanism, the QCC reinforces the reliability and comparability of Kor-GLASS–generated AMR data reported in phase-based national surveillance analyses covering Phases I [14] and II [15]. This internal verification framework also underpins the credibility of species-specific studies based on Kor-GLASS-generated data, including AST-focused studies on *Streptococcus pneumoniae* [16] and *Enterococcus faecium* [17], as well as a study on emerging carbapenemase-producing *Pseudomonas aeruginosa* [18].

AST is inherently susceptible to variability related to result interpretation, particularly for isolates with results near established clinical breakpoints. Previous proficiency testing studies have demonstrated that such discrepancies commonly arise from interpretive inconsistency, delayed adoption of updated guidelines, and limitations in standardization rather than intrinsic methodological failure [19, 20]. In the present study, major errors observed during Phase II were primarily attributable to interpretation-related issues, including discrepancies near breakpoints (Table 3). Following targeted corrective actions, including focused education, repeat testing, and refinement of data handling procedures, these error categories were substantially reduced in Phase III. Given that small analytical or interpretive deviations in AST may lead to categorical discrepancies near clinical breakpoints, the observed improvements support the interpretation that structured, feedback-driven quality assurance interventions may help reduce interpretive variability in AST and contribute to sustained performance within national antimicrobial resistance surveillance systems.

Phase III of the Kor-GLASS program was marked by the introduction of *Haemophilus* spp. as an additional target organism and the incorporation of ceftazidime-avibactam into the AST panel. A previous report comparing AST methods for ceftazidime-avibactam highlighted the need for thorough validation [21]. AST for *Haemophilus* spp. is technically challenging because it requires specific media and AST panels [22], and the emergence of β-lactamase-negative ampicillin-resistant *Haemophilus influenzae* has increased the complexity of routine testing in clinical laboratories [23]. Because the addition of new bacterial species or antimicrobial agents may introduce unrecognized sources of variability, verification of analytical performance during such transitions is essential. In this study, interlaboratory validation for *Haemophilus* spp. (Table 5) and performance assessment of ceftazidime-avibactam susceptibility testing (Table 4) were conducted, demonstrating acceptable and consistent results. These findings underscore the importance of structured validation processes accompanying system updates to ensure continuity and reliability of surveillance data during program expansion.

Several previous studies have utilized GLASS-aligned frameworks to evaluate AMR surveillance systems and resistance trends. An assessment of GLASS implementation in the WHO Eastern Mediterranean region revealed that while the system facilitated region-wide AMR data generation, further efforts are required to strengthen data quality and long-term system sustainability [24]. A large-scale cross-national analysis identified a statistically significant positive correlation between national antimicrobial consumption levels and resistance rates in major pathogens, underscoring the importance of integrated surveillance for effective antimicrobial stewardship [25]. In addition, evidence from the South and South-East Asian region demonstrated that a structured international EQA network can systematically improve laboratory diagnostic performance and data reliability for GLASS-aligned AMR surveillance [26]. In contrast to these studies, the present study focuses on the operational performance of quality assurance itself and demonstrates that a centralized, QCC-led framework with continuous IPT, EQA, and corrective actions can sustain high analytical performance during a phase transition involving system expansion.

As antimicrobial resistance patterns continue to evolve [27], quality assurance activities within the Kor-GLASS framework should be sustained and periodically refined in alignment with global updates and international guidance, including the GLASS recommendations. In parallel, transparent reporting of clinical microbiology data, as highlighted by the Microbiology Investigation Criteria for Reporting Objectively (MICRO) framework, may further support the interpretation and comparability of surveillance-based AMR data [28]. Continuous adaptation of quality assurance strategies is essential to maintain the reliability and comparability of surveillance data as new bacterial species and antimicrobial agents are incorporated into routine monitoring. In addition, the anticipated introduction of whole-genome sequencing into Kor-GLASS surveillance underscores the need to proactively establish genomic quality assurance frameworks. International genomic surveillance networks have demonstrated that interlaboratory variation and data quality issues can substantially affect the reliability of WGS-based AMR surveillance [29]. Strengthening quality assurance across both phenotypic and genomic approaches will be critical for maintaining the long-term robustness of national AMR surveillance.

This study has several limitations inherent to surveillance-based quality assurance frameworks. First, the intensity of IPT-based quality assurance differed across bacterial species because the number of eligible clinical isolates varied substantially by organism. As a result, high-volume pathogens were evaluated with greater statistical robustness, whereas conclusions for less frequently isolated species were necessarily based on more limited observations. Relatedly, several target organisms with extremely low isolation frequencies, such as *Shigella* spp. and *Neisseria* spp., were not included in IPT, thereby limiting the comprehensiveness of quality assurance coverage. Second, while a marked reduction in major errors, particularly those related to AST reading, was observed following educational interventions and feedback coordinated by the QCC, the present study was not designed to formally establish a causal relationship between specific corrective actions and performance improvement. Accordingly, the observed improvements should be interpreted as temporal associations within a continuous quality assurance process rather than as definitive evidence of causality. Finally, because the Kor-GLASS framework operates within a centralized national surveillance infrastructure supported by standardized governance, coordinated quality assurance activities, and dedicated laboratory resources, the present findings may not be directly generalizable to settings with different healthcare or laboratory capacities.

In conclusion, this study shows that a centralized, QCC-led quality assurance framework can maintain stable and reliable AMR surveillance performance during a period of system expansion. By systematically evaluating IPT and EQA outcomes, this study positions quality assurance activities not only as routine operational monitoring but also as an evidence-based evaluation of how standardized laboratory data are generated and validated within a surveillance system. These findings underscore the importance of maintaining centrally coordinated quality assurance as a core structural element of AMR surveillance and its future development. This work represents an advance in biomedical science because it demonstrates that centrally coordinated quality assurance can sustain reliable AMR surveillance performance.

## Summary table

### What is known about this subject

1. National AMR surveillance depends on standardized laboratory testing and robust quality assurance systems.
2. Interlaboratory variability in AST interpretation can compromise the comparability of surveillance data.
3. System expansion with new pathogens or antimicrobials may introduce additional analytical variability.

### What this paper adds

1. A centralized, QCC-led framework sustained high IPT and EQA performance.
2. Targeted corrective actions reduced interpretation-related major errors across participating centers.
3. Structured validation ensured reliable introduction of new species and agents for IPT.

## Data Availability

The original contributions presented in the study are included in the article/Supplementary Material, further inquiries can be directed to the corresponding author.
